# Role of Calcr expressing neurons in the medial amygdala in social contact among females

**DOI:** 10.1186/s13041-023-00993-4

**Published:** 2023-01-19

**Authors:** Kansai Fukumitsu, Arthur J. Huang, Thomas J. McHugh, Kumi O. Kuroda

**Affiliations:** 1 Laboratory for Affiliative Social Behavior, RIKEN Center for Brain Science, Hirosawa 2-1, Wakoshi, Saitama 351-0198 Japan; 2 Laboratory for Circuit and Behavioral Physiology, RIKEN Center for Brain Science, Wakoshi, Saitama 351-0198 Japan

**Keywords:** Calcitonin receptor, Medial amygdala, Social contacts, RNA interference

## Abstract

Social animals become stressed upon social isolation, proactively engaging in affiliative contacts among conspecifics after resocialization. We have previously reported that calcitonin receptor (Calcr) expressing neurons in the central part of the medial preoptic area (cMPOA) mediate contact-seeking behaviors in female mice. Calcr neurons in the posterodorsal part of the medial amygdala (MeApd) are also activated by resocialization, however their role in social affiliation is still unclear. Here we first investigated the functional characteristics of MeApd Calcr + cells; these neurons are GABAergic and show female-biased Calcr expression. Next, using an adeno-associated virus vector expressing a short hairpin RNA targeting Calcr we aimed to identify its molecular role in the MeApd. Inhibiting Calcr expression in the MeApd increased social contacts during resocialization without affecting locomotor activity, suggesting that the endogenous Calcr signaling in the MeApd suppresses social contacts. These results demonstrate the distinct roles of Calcr in the cMPOA and MeApd for regulating social affiliation.

**Supplementary Information:**

The online version contains supplementary material available at 10.1186/s13041-023-00993-4.

## Introduction

Social animals proactively seek contact with conspecifics, exhibiting stress responses upon isolation. Specific neurons in various brain regions, such as dopaminergic neurons in the dorsal raphe [1], parvocellular oxytocin neurons in the paraventricular nucleus of the hypothalamus [2], and Tac1-positive neurons in the medial amygdala (MeA) [3] have been implicated in the regulation of social contact and prosocial behaviors, suggesting that a brain network including these areas governs socialization in mammals.

We have recently identified that the calcitonin receptor (Calcr) and amylin, its brain ligand, in the medial preoptic area (MPOA), are required to seek and sense social contacts [4]. Calcr is a seven-transmembrane G-protein-coupled receptor involved in calcium homeostasis through its ligand calcitonin in the periphery. However, in the brain, Calcr directly binds with amylin, a 37-aa pancreatic peptide hormone, activating the calcitonin receptor complex, consisting of Calcr and a receptor activity-modifying protein 1–3 (RAMP1-3) [5]. Further, Calcr + neurons in the central part of the MPOA (cMPOA) are critical for parental behavior in mice [6] and amylin expression in the MPOA is inhibited by social isolation in adult female mice and recovered following reunion with peers [4]. This demonstrated that amylin-expressing cells in the MPOA are constitutively active during group housing conditions but deactivated by acute social isolation, even if the peers are in the same cage but separated by a windowed partition from a single subject mouse (Fig. 2A, somatic isolation hereafter). Somatically isolated mice explore the partition by sniffing and rearing, vigorously biting the partition only when the peers are present beyond it; thus, this partition-biting behavior is deemed as contact-seeking behavior to maintain affiliative contacts among female mice. Upon returning the females to group housing (reunion) by replacing the windowed partition with a sham partition with a large entrance, the isolated animal engages in affiliative contact such as crawling-under, eventually forming a huddle. Genetic and pharmacogenetic methods revealed that Calcr in the MPOA mediates both contact-seeking and contact behaviors while amylin specifically mediates contact-seeking behaviors.

In our previous study, we indentified Calcr expressing neurons in the MeA which, similar to Calcr + neurons in the MPOA, were activated by social interactions among females [4]. As the MeA is an important site mediating various social behaviors, not only prosocial allogrooming but also intermale aggression and mating behaviors [7–9], we hypothesized that MeA Calcr + neurons may play a role in behavioral regulation during social isolation and contact. Here, we aimed to investigate the general features of Calcr + neurons in the MeA and Calcr function in the MeA during social isolation and reunion using RNA interference.

## Materials and methods

### Animals

All animal experiments were performed in accordance with the guidelines of RIKEN’s Animal Experiment Committee and conducted in accordance with the National Institutes of Health guidelines (NIH publication no. 85–23, revised 1985).

BALB/c mice were originally purchased from the Jackson Laboratory in Japan and raised in our breeding colony. Mice were maintained under controlled conditions [12 h:12 h light/dark cycle (lights on at 08.00 am) with food and water ad libitum]. BLAB/c mice of both sexes were housed in groups of four or five after weaning at 4 weeks. All the mice were 3–7 months old at the beginning of the experiments.

### Behavioral experiments

The social isolation and reunion test was performed as described previously (Fig. 2A; [4]). Briefly, four group-housed BALB/c adult female mice were placed in a separation apparatus in the middle of the test cage consisting of a replaceable sham partition with two large holes and were accustomed to the cage for 7 days. Then, one out of four mice was separated into a no-nest chamber by replacing the sham partition with a real one with two barrier windows (somatic isolation). Three cage mates remained in the same chamber as their nest (3-together). Two days after somatic isolation, the real partition was replaced by a sham partition, and the pre-isolated mouse was reunited with its cage mates. Behavioral changes after somatic isolation or reunion were tracked for > 2 h and scored during either 15-s or 5-min intervals based on a previously established ethogram [4]. In addition to direct physical contact, the following behaviors were coded: (1) general movement (still/movement); (2) exploratory behavior (partition-sniffing, rearing, and digging); (3) contact-seeking behavior (partition-biting); (4) nonsocial behaviors (self-grooming, eating, and panic); (5) social behavior (crawling-under, peer-sniffing, allogrooming, mounting, peer-biting, and chasing). All behavioral data was quantified in an experimenter-blind setting.

### Histochemistry and image acquisition

Detailed protocols for in situ hybridization (ISH) and immunohistochemistry (IHC) are described elsewhere [4, 10]. For ISH combined with Calcr IHC, *GAD67* mRNA (1064–2045 bp, NM_008077) was detected by ISH and Calcr-ir (immunoreactive) cells were detected by 3,3'-diaminobenzidine (DAB) reaction without nickel.

For Calcr or amylin mapping experiments, amygdala sections were stained with anti-Calcr or anti-amylin antibodies; positive cells were detected with a DAB reaction with nickel. Following the initial staining, NeuN-ir cells were counterstained with ImmPACT Vector Red (Vector Laboratories). For double labeling of Calcr and ERα, Calcr-ir neurons were detected with a DAB reaction without nickel. Following the first staining, ERα+ cells were detected by a DAB reaction with nickel.

To test the knockdown efficacy of Calcr shRNA, Calcr-ir neurons were detected by DAB reaction with nickel. Following the first staining, shRNA-viral infected and EGFP-ir cells were detected with ImmPACT Vector Red.

The ICH antibodies were as follows: rabbit anti-Calcr (1:4,000, PAb188/10, Welcome receptor antibodies); rabbit anti-amylin (1:20,000, cat#H-017-11, Phoenix Pharmaceuticals, Burlingame, CA, USA); rabbit anti-GFP antibody (1:5,000, 598, MBL); rabbit anti-ERα (1:20,000, 06–935, Merck Millipore), biotin-conjugated horse anti-rabbit (1:2000, BA-1100, Vector Laboratories), or anti-mouse (1:2000, BA-2000, Vector Laboratories) secondary antibody.

### Image acquisition and analysis

Bright-field images were acquired using a NanoZoomer Digital Pathology (Hamamatsu Photonics) with a 20 × objective.

**Fig. 1:**
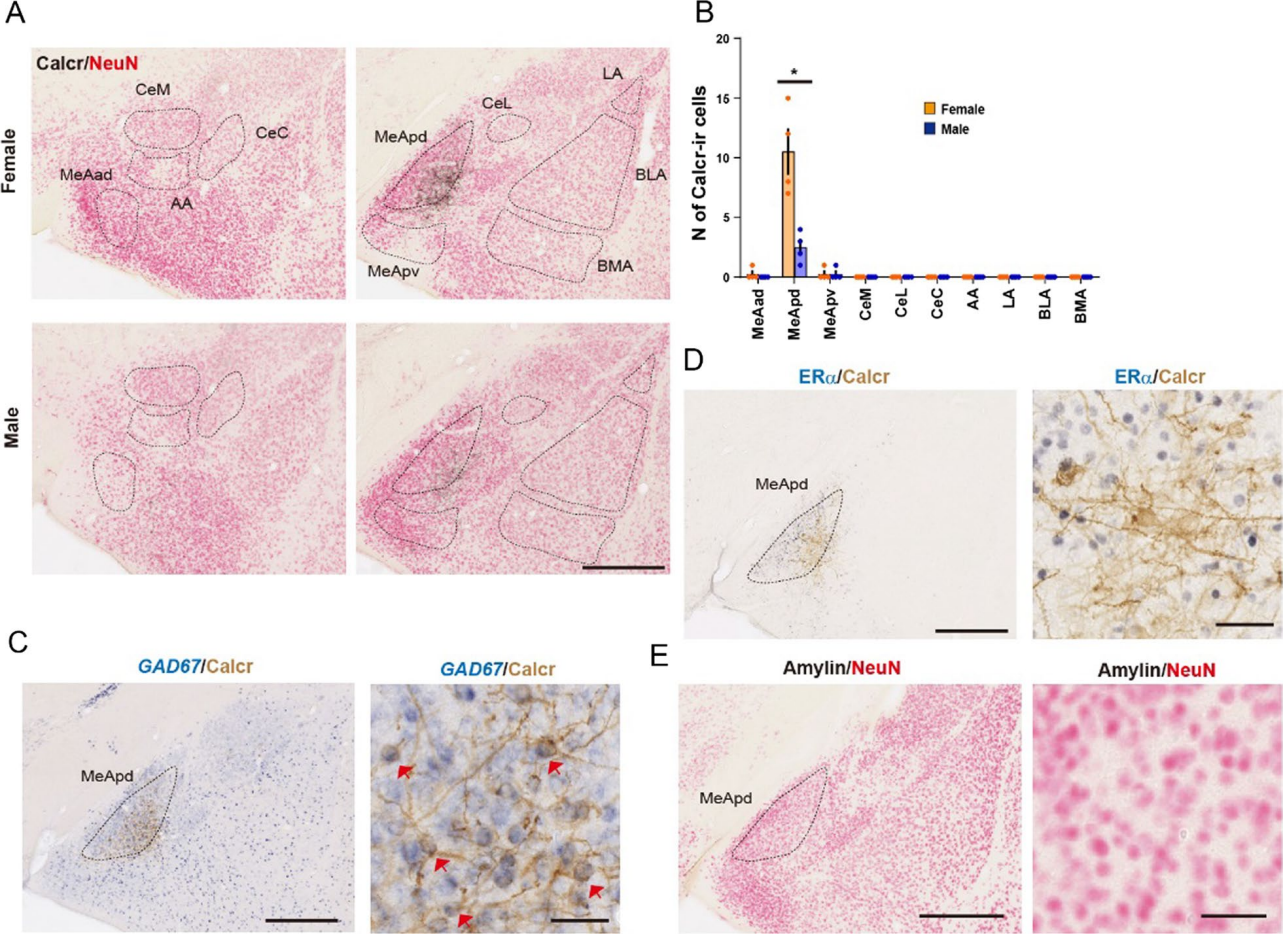
Sexual dimorphic Calcr expression and gene profiles of Calcr + cells in the medial amygdala. **A** Representative coronal brain sections of both sexes, showing the distribution of Calcr immunoreactivity (Calcr-ir, black) and NeuN counterstaining (red) in subregions of the amygdala in female or male mice. *MeAad* anteroventral part of the medial amygdala, *MeApd* posterodorsal part of the medial amygdala, *MeApv* posteroventral part of the medial amygdala, *CeM* medial part of the central amygdala, *CeL* lateral part of the central amygdala, *CeC* capsular part of the central amygdala, *AA* anterior amygdaloid area, *LA* lateral amygdala, *BLA* basolateral amygdala, *BMA* basomedial amygdala. Scale bars, 500 μm. **B** Number of Calcr-ir neurons in each subregion of female or male mice (n = 4 mice per group). **C** Sections stained with *Gad67* mRNA (blue) by ISH together with anti-Calcr (brown) antibody. Arrowheads indicate double-labeled cells. Scale bars, 500 μm (left) and 50 μm (right). **D** Sections stained with anti*-*ERα (black) by IHC together with anti-Calcr (brown) antibody. Scale bars, 500 μm (left) and 50 μm (right). **E** Sections stained with anti*-*amylin (black) by IHC together with anti-NeuN (red) antibody. Scale bars, 500 μm (left) and 50 μm (right). Asterisks in (**B**) denote significant differences between two groups (Welch’s unpaired t-test, *P < 0.05). Graphs represent mean ± SEM. See Additional file 1 for detailed statics

For Calcr mapping in the amygdala, conservative contours were set on brain coronal sections based on NeuN counterstaining as previously described [4]. The number of labeled somata of each conservative contour was manually counted using the ImageJ plugin “Cell Counter.” A single brain section from each mouse was used for histological analyses related to Fig. 1. Histological analyses were not blinded due to the high complexity of these tasks.

### Stereotactic injection and gene knockdown

Stereotactic AAV injections were performed as previously [4, 6, 10]. Stereotactic coordinates for the MeApd were obtained from the mouse brain atlas [11]. The coordinates used were as follows: MeApd (AP − 1.6, ML ± 2.4, DV − 5.15).

**Fig. 2:**
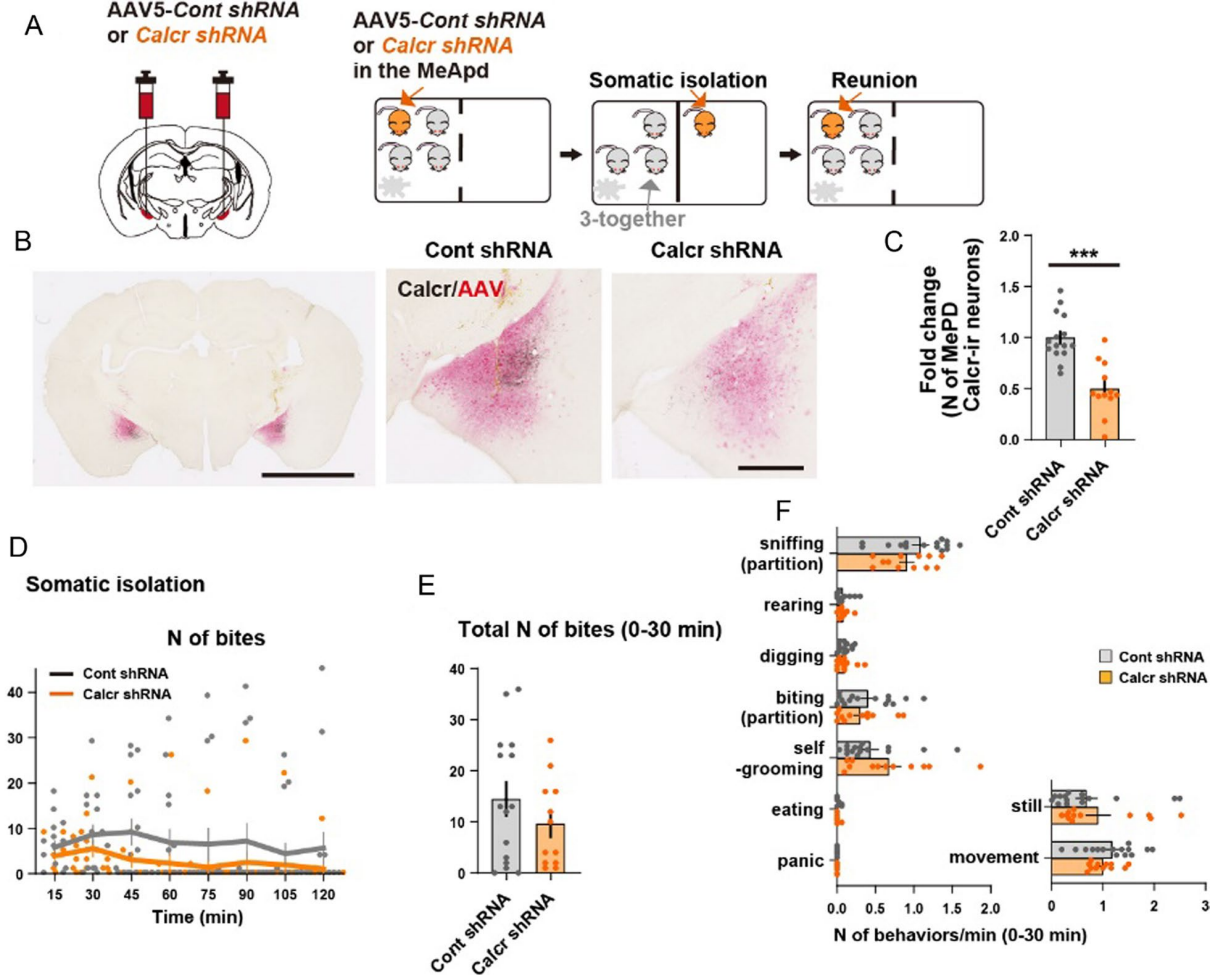
Targeted *Calcr* knockdown in the MeApd does not affect behavioral responses to social isolation. **A** Timeline of behavioral experiments. At 2 weeks before testing, female mice were administered an AAV expressing shRNA-EGFP for specific *Calcr* knockdown or with a shRNA virus carrying scrambled shRNA (Cont shRNA) into the MeApd region. An AAV-injected adult female mouse was subjected to a somatic isolation test and reunion tests. **B** Representative coronal sections of shRNA-EGFP viral expression in the MeApd showing the efficacy of *Calcr shRNAs*. Scale bars, 2.5 mm (Left), 500 μm (Middle and Right). A shRNA virus carrying scrambled shRNA was used as control. **C** Quantification of fold changes in the number of Calcr + cells distributed in the MeApd (Cont shRNA: n = 15 mice and Calcr shRNA: n = 12 mice). **D** Time course of biting responses (Cont shRNA: n = 15 mice and Calcr shRNA: n = 12 mice). **E** Quantification of the total number of biting responses over 30 min (Cont shRNA: n = 15 mice and Calcr shRNA: n = 12 mice). **F** Number of various behaviors per min observed under somatic isolation for 30 min (Cont shRNA: n = 15 mice and Calcr shRNA: n = 12 mice). Time bins 15 s (**D**–**F**). Asterisks denote significant differences between the two groups (**C**, **E**, **F**: Welch’s unpaired t-test, **D**: 2-way repeated measure ANOVA tests followed by Sidak's post hoc tests; ***P < 0.001). Graphs represent mean ± SEM. See Addi for detailed statics

The AAVs AAV5-*Calcr shRNA-CAG-EGFP* and AAV5-*Cont shRNA-CAG-EGFP* (1.0E11-1.0E12 vg/mL) were those used in our previous publication [4], and provided by Thomas McHugh’s laboratory. A total of 100 nL of each AAV was injected bilaterally into the MeApd of one in four female mice. At 2 weeks after AAV injection, viral injected mice were subjected to a somatic isolation test and a reunion test. After behavioral testing, mice were evaluated for proper viral injections: briefly, the brains were coronally sectioned at 40 μm, and every third section was taken out as one series. In one series, nine consecutive sections covering the MeApd were doubly stained for Calcr and EGFP immunohistochemistry. Mice without any expression of EGFP in the MeApd were excluded from the analyses (1 out of 16 females injected with Control shRNA and 4 out of 16 females). We confirmed no Calcr immunoreactivity in the EGFP + neurons as in our previous study [4]. Then, all the MeApd Calcr + neurons in the nine coronal sections were manually counted to yield Fig. 2C.

### Statistical analysis

Statistical analyses were conducted by unpaired t-test in Microsoft Excel for single comparisons and repeated 2-way ANOVA with multiple comparison test using GraphPad Prism8 software (GraphPad) with a 95% confidence limit. In addition to constructing a Q-Q plot, we used the D'Agostino-Pearson normality test to assess the normality of distribution. Data are indicated as mean ± SEM. All data visualization was conducted using GraphPad Prism8 software.

## Results

### Specific localization and cellular properties of Calcr in the MeApd

We first counted the number of Calcr-ir cells in the MeA, as well as in the other major amygdala subregions, of both sexes. The MeA was anatomically subdivided into anteroventral, anterodorsal (MeAad), posterodorsal (MeApd), and posteroventral (MeApv) parts. Calcr expression was confined to the posterodorsal part of the medial amygdala (MeApd) of BALB/c mice irrespective of sex (Fig. 1A). Within the MeApd, there was more Calcr + cells localized in the lateral part than in the medial part. Moreover, Calcr expression in the MeApd was significantly higher in females than in males, indicating female-biased Calcr expression in the MeApd (Fig. 1B). As Calcr expression in the MeApd was low in males, hereafter we focused on females.

Next, we examined the molecular profile of Calcr + cells in the female MeApd. We previously found that Calcr + neurons can be either glutamatergic (expressing *VGlut2* mRNA) or GABAergic (expressing *Gad67* mRNA) in the MPOA [4, 6]. In the MeApd, all the Calcr-ir cells co-expressed a GABAergic (Gad67) neuronal marker (Fig. 1C). Further, in the MPOA roughly two thirds of Calcr-ir cells express estrogen receptor alpha (ERα) (Additional file 1: Fig. S1A) [6]. In the MeApd however, Calcr-ir cells did not co-express ERα (Fig. 1D). Moreover, we did not observe any amylin + neurons in the MeApd (Fig. 1E). This result suggests that the activity of Calcr in the MeApd is regulated in an amylin-independent manner as Calcr is constitutively active in the absence of agonists [12]. Another possibility is that amylin + cells in the MPOA, arcuate nucleus or other areas project to the MeApd and regulate the function of Calcr + cells in the MeApd.

### Knocking down Calcr expression in the MeApd promotes behavioral responses to social reunion

*Calcr* gene knockout in mice produces embryonic lethality [13] and Calcr^fl^°^x^ mice exhibit mildly altered social behaviors (data not shown), thus similar to our previous work we utilized an AAV-mediated short hairpin interference RNA (shRNA), namely AAV5-*hH1-shCalcr-CAG-EGFP,* to knockdown Calcr in the MPOA and an AAV5-*hH1-Scrambled-CAG-EGFP* as control [4, 6] to address if the Calcr in the MeApd is required for the behavioral response to social isolation and/or reunion.

BALB/c adult female mice received Calcr shRNA or Control shRNA AAV injections to the bilateral MeApd and their social behavior was examined (Fig. 2A). We retrospectively confirmed that the shRNA targeting *Calcr*, but not the scrambled control RNA, significantly reduced Calcr expression in the MeApd at 16 days after injection, observing a 50.2% reduction in Calcr + neurons (Fig. 2B, C). Consistent with this, IHC staining revealed that the MeApd injected with the control shRNA expressed endogenous Calcr gene, while MeApd -neurons expressing shRNA did not express endogenous Calcr, confirming the shRNA efficiently suppressed Calcr expression in the MeApd.

Two weeks after AAV injection, the mouse responses to somatic isolation were examined (Fig. 2D–F). In contrast to Calcr knockdown in the cMPOA, neither partition-biting nor sniffing or rearing around the partition were significantly altered by Calcr knockdown in the MeApd (Fig. 2D–F).

**Fig. 3:**
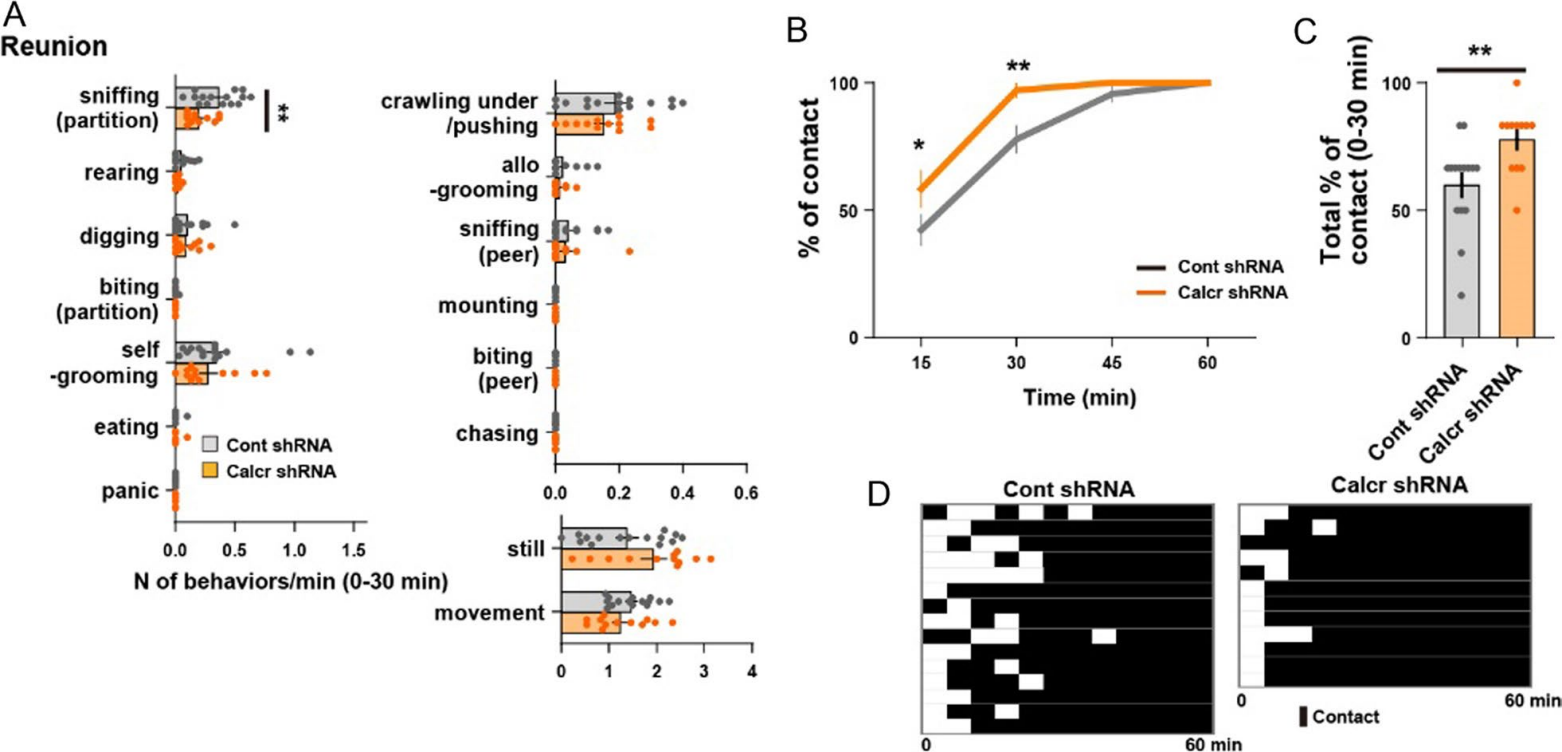
Targeted *Calcr* knockdown in the MeApd facilitates behavioral responses to the reunion. **A** Number of various behaviors per min observed under social reunion for 30 min (Cont shRNA: n = 15 mice and Calcr shRNA: n = 12 mice). **B** Time course of the percentage of social contacts (Cont shRNA: n = 15 mice and Calcr shRNA: n = 12 mice). (**C**) Total percentage of social contacts over 30 min (Cont shRNA: n = 15 mice and Calcr shRNA: n = 12 mice). **D** Raster plots showing the effects of Calcr knockdown in the MeApd on contact occurrence. Time bins 15 s (**A**), 5 min (**B**–**D**). Asterisks denote significant differences between the two groups (**A**, **C**: Welch’s unpaired t-test, **B**: 2-way repeated measure ANOVA tests followed by Sidak's post hoc tests; *P < 0.05, **P < 0.01). Graphs represent mean ± SEM. See Additional file 1 for detailed statics

Two days after somatic isolation, the mice were subjected to a social reunion test (Fig. 3A–D). We have previously reported that isolated control mice show direct social contact behaviors, such as crawling-under, peer-sniffing, and allogrooming toward peers. After this active phase, the mice gradually calm down and sleep in a cluster, designated as sleep huddle (Fig. 3B, D) [4]. Behavioral analysis showed that Calcr knockdown in the MeApd inhibited sniffing behaviors around the partition, although affiliative social behaviors, such as crawling-under and allogrooming were not affected (Fig. 3A). On the other hand, Calcr knockdown in the MeApd mildly increased direct contacts during the active phase (Fig. 3B–D). These findings indicate that Calcr molecular expression patterns in the MeApd are functionally distinct from those in the cMPOA, and do not appear to mediate affiliative contact-seeking or contact either during somatic isolation or reunion.

## Discussion

The MeApd regulates female-specific behaviors, as well as male specific social behaviors such as male sexual behavior and partner preference [14–16]. For instance, optogenetic activation of inhibitory neurons in the MeApd promotes parental behavior (pup-grooming) in female mice [17] and aromatase-expressing cells in the MeApd mediate maternal aggression [18]. In the present study, we found a female-biased expression of Calcr in the MeApd; moreover, Calcr in the MeApd appears to mediate social contact behaviors in female mice.

The MeA and the MPOA share interesting features, such as sexual dimorphism, and play pivotal roles in regulating social behavior. Developmentally, these shared features are supported by the fact that GABAergic neurons in the MeA are born in the MPOA [19]. The number of neurons and regional volume in the SDN-POA (sexually dimorphic nucleus of the preoptic area) and the MeA are higher in male than in female mice [20, 21]. These morphological sex differences appear related to a sex-biased addition of newly born cells during puberty [22]. In addition to these anatomical differences, recent single-cell transcriptomic analyses have identified molecular sex differences such as Brs3 and Esr1 expression in the MPOA and MeA [17, 23, 24]. ERα is abundantly expressed in the MeA as well as the MPOA in both sexes, but higher in female mice [23, 25]. Previous reports showed that ERα in the MeA does not appear to regulate male sexual and aggressive behavior in adulthood [26], but rather is required to masculinize the pubertal neural circuitry responsible for these behaviors [21]. The MeA ERα also plays an important role in body weight control by stimulating physical activity in female and male mice [26, 27]. However, MeApd Calcr + cells did not colocalize with ERα (Fig. 1D), and the targeted Calcr knockdown in the MeApd did not significantly alter locomotor activity or body weight (Fig. 3A, Additional file 1: Fig. S1B, C).

In the present study, we found that Calcr + neurons in the MeA are GABAergic, suggesting that they may derive from Calcr + neurons in the MPOA and share functional properties with them. Nevertheless, the function and properties of Calcr + neurons in the MeA are distinct from those in the MPOA. During somatic isolation, Calcr neurons in the MPOA facilitate affiliative contact-seeking during somatic isolation, but in the MeA, these cells do not appear to be involved in contact-seeking behaviors. Moreover, during knocking down Calcr in the cMPOA reduces social contact behaviors such as crawling-under [4], while Calcr knockdown in the MeA increases contact behaviors. This finding can be interpreted in two ways: one possibility is that Calcr + neurons in the MeA directly inhibit social contact behavior via Calcr. The apparently opposite functions of the cMPOA (facilitatory) and MeA (inhibitory) on social contact may be partially consistent with previous findings that MeA neurons suppress maternal behaviors in virgin female rats and promote infanticide in male mice [17, 28], while cMPOA neurons facilitate maternal and paternal behaviors. The efferent projections from the MeA are directed strongly to the MPOA [29], including the cMPOA Calcr + neurons [6], suggesting that the MeApd modulates the function of MPOA Calcr + cells. cMPOA Calcr + neurons also send modest projections to the MeApd. Further studies should clarify possible anatomical and functional connections between Calcr + neurons in the MeApd and cMPOA. Another possibility is that MeApd Calcr + neurons may be involved in the perception of environmental threats; in this case, Calcr knockdown may decrease sensitivity to the partition change and thus reduce partition sniffing (Fig. 3A), allowing more contacts by peer mice. Further research is needed to distinguish these possibilities. Nevertheless, the present results suggest that MeApd Calcr neurons do not positively mediate social contact behaviors such as crawling-under and allogrooming, even if other neurons in the MeA may mediate social contact behaviors.

The MeA relays chemosensory information from the main olfactory bulb and accessory olfactory bulb (AOB) to medial forebrain regions such as the MPOA. In male rats, the MeApd is activated in response to the nonvolatile odor of estrous female rats [30]. The number of c-Fos + cells increases in the MeApd of female mice exposed to the male pheromone ESP1 [31]. In the MeApd, there is a cluster of ERα + cells in the medial part close to the optic tract, while the cluster of Calcr + cells is present in the more lateral part (Fig. 1A, D), the same region in which the number of c-Fos-ir cells increases following social reunion (Additional file 1: Fig. S1D). This differential distribution of active neurons within the MeApd has been reported previously in male rats [32]. The lateral portion of the MeApd appears to be activated by somatosensory signals during copulation, while the medial portion of the MeApd appears to be activated by chemosensory cues from female rats. Since axon terminals from AOB are found in the medial part of the MeApd [33], these findings suggest that MeApd Calcr + cells may be activated by somatosensory inputs from direct female-female social interaction such as anogenital sniffing, but not female pheromones. This result is consistent with our previous study in which amylin-Calcr signaling in the MPOA was not activated by chemosensory cues [4]. Future research is warranted to elucidate the activation mechanisms of Calcr + neurons in the MeApd.

The limitation of this study is the partial reduction of Calcr expression by shRNA due to the infection efficacy of AAV serotype 5 (AAV5) as suggested [4]. Using higher infection efficient AAV serotypes such as DJ/8, or conditional knockouts using Calcr-flox mice may achieve a higher reduction in Calcr + neurons and reach a significant change in biting responses upon acute social isolation.

## Supplementary Information

**Additional file 1: Fig. S1.** Supportive experiments for this article and statistical analysis.

## Data Availability

The materials generated in this study are available from the corresponding author upon reasonable request with a completed Materials Transfer Agreement.

## Abbreviations

Calcr: Calcitonin receptor
RAMP: Receptor activity-modifying protein
NIH: National Institutes of Health
ISH: In situ hybridization
IHC: Immunohistochemistry
DAB: 3,3'-Diaminobenzidine
MPOA: Medial preoptic area
cMPOA: Central part of the medial preoptic area
MeA: Medial amygdala
MeAad: Anterodorsal part of the medial amygdala
MeApd: Posterodorsal part of the medial amygdala
MeApv: Posteroventral part of the medial amygdala
CeM: Medial part of the central amygdala
CeL: Lateral part of the central amygdala
CeC: Capsular part of the central amygdala
AA: Anterior amygdaloid area
LA: Lateral amygdala
BLA: Basolateral amygdala
BMA: Basomedial amygdala
ERα: Estrogen receptor alpha
shRNA: Short hairpin interference RNA
AAV: Adeno-associated virus
SDN-POA: Sexually dimorphic nucleus of the preoptic area
AOB: Accessory olfactory bulb
