# Supplementary material for: Role of Calcr expressing neurons in the medial amygdala in social contact among females
Source: Mol Brain. 2023 Jan 19;16:10. doi: 10.1186/s13041-023-00993-4 (PMC9850531; doi:10.1186/s13041-023-00993-4)
Supplement: Supplementary file 1 — Additional file 1: Fig. S1. Supportive experiments for this article and statistical analysis. [file 13041_2023_993_MOESM1_ESM.docx]

**Supplementary information for**

Role of Calcr expressing neurons in the medial amygdala in social contact among females

Kansai Fukumitsu*, Arthur J. Huang, Thomas J. McHugh, and Kumi O. Kuroda

***Corresponding author:** Kansai Fukumitsu

Laboratory for Affiliative Social Behavior, RIKEN Center for Brain Science

Hirosawa 2-1, Wakoshi, Saitama 351-0198, Japan

E-mail: [kansai.fukumitsu@riken.jp](mailto:kansai.fukumitsu@riken.jp), Tel: +81-48-467-7556, Fax: +81-48-467-6853

**This PDF file includes:**

Fig. S1 and Statistical analysis

## **Supplementary Figure**


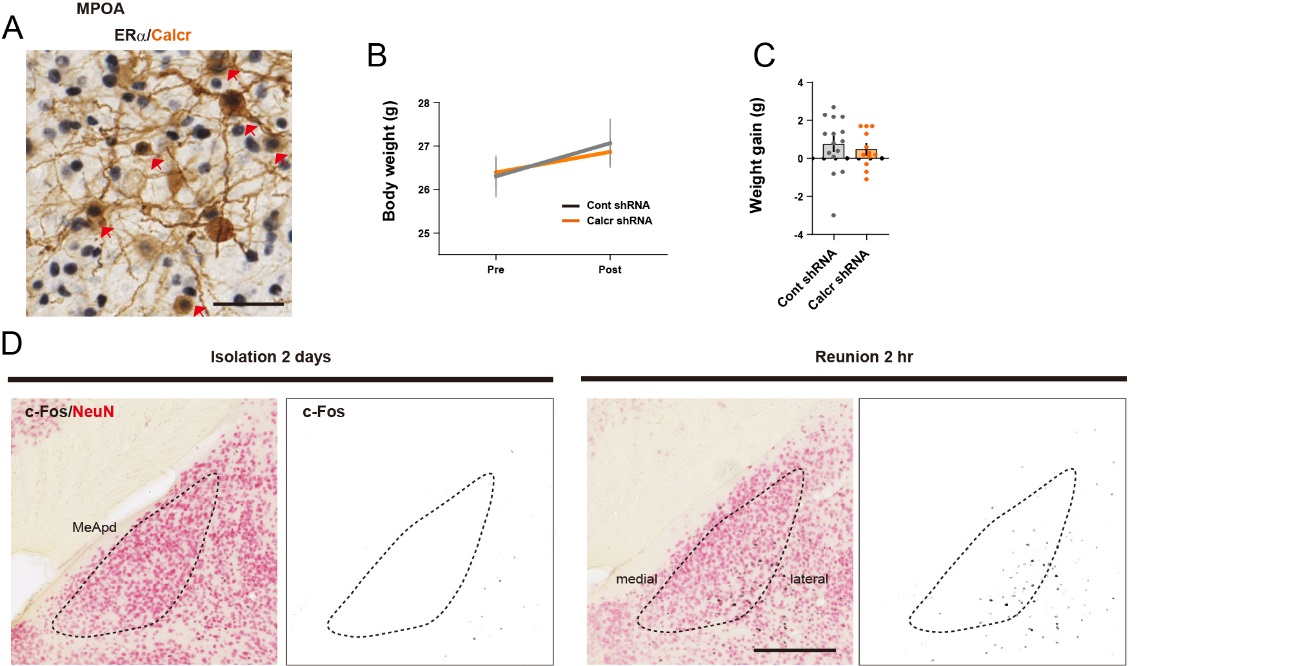


**Supplementary Figure 1 Supportive experiments for this article**

(**A**) Sections including MPOA were stained with *anti-*ERα (black) in combination with anti-Calcr (brown) antibody by IHC. Arrowheads indicate double-labeled cells in the MPOA. Scale bars, 50 μm.

(**B**, **C**) Targeted knockdown of Calcr in the MeApd does not affect weight gain. Body weight (b) and weight gain (c) recorded on the day of AAV injections (pre) and 1 day after the reunion test (post) (Cont shRNA: n = 15 mice and Calcr shRNA: n = 12 mice). No significant differences between groups [2-way repeated measure ANOVA with Sidak multiple comparison tests for (b). Welch’s unpaired t-test for (c)]. Graphs represent mean ± SEM. See Supplementary data for detailed statics.

(**D**) Distribution of c-Fos-ir cells in the MeApd in the control (2 days of somatic isolation) and 2 hr of reunion. Coronal sections stained for c-Fos (Black) in combination with NeuN (red) in the amygdala. Scale bars, 250 μm.

## **Statistical analysis**

| **Figure** | **Part** | **test** | **t-value with degree of freedom for t-tests, exact P-value with degree of freedom for ANOVAs** |
| --- | --- | --- | --- |
| 1 | B | Two-tailed unpaired t-test | p=0.391, t=1, df=3 (MeAad), p=0.015, t=4.086, df=4 (MeApd),p=1, t=0, df=6 (MeApv),n.a. (CeM, CeL, CeC, AA, LA, BLA, BMA), Welch's unpaired t-test |
| 2 | C | Two-tailed unpaired t-test | p=2.70E-05, t=5.27, df=22 , Welch's unpaired t-test |
| 2 | D | Repeated measure Two-way ANOVA with Sidak's multiple comparison test | F7,175=0.4168, df=7, p=0.891 (interaction term), F1,25=1.875, df=1, p=0.1831 (Treatment), F7,175=1.434, df=7, p=0.1944, Sidak's multiple comparison, p=0.9994 (15 min: Cont shRNA vs Calcr shRNA), p=0.9844 (30 min: Cont shRNA vs Calcr shRNA), p=0.5396 (45 min: Cont shRNA vs Calcr shRNA), p=0.8404 (60 min: Cont shRNA vs Calcr shRNA), p=0.7651 (75 min: Cont shRNA vs Calcr shRNA), p=0.8242 (90 min: Cont shRNA vs Calcr shRNA), p=0.9984 (105 min: Cont shRNA vs Calcr shRNA), p=0.8430 (120 min: Cont shRNA vs Calcr shRNA) |
| 2 | E | Two-tailed unpaired t-test | p=0.244, t=1.193, df=24, Welch's unpaired t-test |
| 2 | F | Two-tailed unpaired t-test | p=0.214, t=1.274, df=25 (sniffing:partition), p=0.863, t=-0.173, df=24 (rearing), p=0.837, t=-0.207, df=19 (digging), p=0.422, t=0.815, df=25 (biting:partition), p=0.210, t=-1.291, df=21 (self-grooming), p=0.750, t=0.321, df=25 (eating), n.a. (panic), p=0.480, t=-0.717, df=23 (still), p=0.261, t=1.153, df=22 (movement), Welch's unpaired t-test |
| 3 | A | Two-tailed unpaired t-test | p=0.00612, t=3.018, df=23 (sniffing:partition), p=0.156, t=1.477, df=18 (rearing), p=0.836, t=0.209, df=25 (digging), p=0.334, t=1, df=14 (biting:partition), p=0.512, t=0.664, df=25 (self-grooming), p=0.877, t=-0.156, df=22 (eating), n.a. (panic), p=0.122, t=-1.601, df=23 (still), p=0.293, t=1.079, df=20 (movement), p=0.385, t=0.882, df=25 (crawling under), p=0.416, t=0.827, df=22 (allo-grooming), p=0.719, t=0.363, df=22 (sniffing:peer), n.a. (mounting), n.a. (biting:peer), n.a. (chasing), Welch's unpaired t-test |
| 3 | B | Repeated measure Two-way ANOVA with Sidak's multiple comparison test | F3,75=2.563, df=3, p=0.061 (interaction term), F1,25=9.836, df=1, p=0.0043 (Treatment), F3,75=63.53, df=3, p<0.0001, Sidak's multiple comparison, p=0.0311 (15 min: Cont shRNA vs Calcr shRNA), p=0.0058 (30 min: Cont shRNA vs Calcr shRNA), p=0.9125 (45 min: Cont shRNA vs Calcr shRNA), p>0.9999 (60 min: Cont shRNA vs Calcr shRNA) |
| 3 | C | Two-tailed unpaired t-test | p=0.0057, t=-3.019, df=25 , Welch's unpaired t-test |
| S1 | B | Repeated measure Two-way ANOVA with Sidak's multiple comparison test | F1,25=0.332, df=1, p=0.569 (interaction term), Sidak's multiple comparison, p=0.989 (Pre: Cont shRNA vs Calcr shRNA), p=0.940 (Post: Cont shRNA vs Calcr shRNA) |
| S1 | C | Two-tailed unpaired t-test | p=0.551, t=0.604 df=24 , Welch's unpaired t-test |
